# The Prognostic Role of the Lung Immune Prognostic Index in Neuroendocrine Prostate Cancer: A Multicenter Retrospective Study

**DOI:** 10.3390/diagnostics16172839

**Published:** 2026-09-03

**Authors:** Merve Turan, Mehmet Nuri Baser, Fatima Ozkaya Kutluay, Ahmet Unlu, Ozlem Kutlu, Umut Cakıroglu, Asim Armagan Aydin, Olcun Umit Unal, Gamze Gokoz Dogu, Esin Oktay

**Affiliations:** 1 Department of Medical Oncology, Faculty of Medicine, Aydin Adnan Menderes University, Zafer Neighborhood, 09100 Aydin, Turkey; mbaser@adu.edu.tr (M.N.B.); esinct@gmail.com (E.O.); 2 Department of Medical Oncology, Faculty of Medicine, Pamukkale University, 20070 Denizli, Turkey; fatimaozkaya@gmail.com (F.O.K.); ggokoz@pau.edu.tr (G.G.D.); 3 Department of Medical Oncology, Antalya Education and Research Hospital, University of Health Sciences, 07100 Antalya, Turkey; md.ahmetunlu@gmail.com (A.U.); drarmaganaydin@gmail.com (A.A.A.); 4 Department of Medical Oncology, Izmir City Hospital, University of Health Sciences, 35540 Izmir, Turkey; ozlemacikerkutlu@gmail.com (O.K.); drumutcakiroglu@gmail.com (U.C.); drolcun@gmail.com (O.U.U.)

**Keywords:** neuroendocrine prostate cancer, Lung Immune Prognostic Index, LIPI, systemic inflammation, overall survival, prognostic biomarker

## Abstract

**Background**: Neuroendocrine prostate cancer (NEPC) is a rare and aggressive malignancy with limited prognostic tools. The Lung Immune Prognostic Index (LIPI), derived from dNLR and lactate dehydrogenase, has demonstrated prognostic value in small-cell lung cancer but has not been evaluated in NEPC. This study assessed the prognostic role of LIPI in NEPC. **Methods**: This multicenter retrospective study included 34 patients with NEPC (21 secondary, 13 de novo) from four centers in Turkey. Laboratory data were collected at NEPC diagnosis (T3), initial prostate cancer diagnosis (T1), and castration-resistant prostate cancer diagnosis (T2). LIPI was scored using original fixed cut-offs. Survival analyses included Kaplan–Meier, Cox regression, and ROC methods. **Results**: At NEPC diagnosis, 18 patients (52.9%) had Good LIPI and 16 (47.1%) Intermediate + Poor LIPI. Intermediate + Poor LIPI was associated with significantly shorter overall survival (median 4 vs. 15 months; log-rank *p* = 0.001; HR 3.83, 95% CI 1.65–8.90). In multivariable analysis, albumin was an independent predictor (HR 0.37, *p* = 0.015), while LIPI showed a trend (HR 2.35, *p* = 0.091). LIPI demonstrated the highest discriminatory ability for 6-month overall survival (AUC 0.763, *p* = 0.009). LIPI at earlier disease stages did not predict time to transformation or castration resistance. Among secondary NEPC patients, worsening LIPI trajectory was associated with shorter survival (median 4 vs. 10 months; log-rank *p* = 0.031). **Conclusions**: LIPI at NEPC diagnosis was associated with overall survival and demonstrated the highest discriminatory ability among the inflammatory indices assessed. As a routine blood-based score, LIPI may support risk stratification at NEPC diagnosis. Prospective validation is warranted.

## 1. Introduction

Among men, prostate cancer ranks as one of the most frequently diagnosed solid tumors globally. Although most patients initially respond to androgen deprivation therapy, neuroendocrine prostate cancer (NEPC) represents a particularly aggressive subtype and is becoming an increasing clinical concern. De novo NEPC is rare, accounting for fewer than 2% of all prostate cancer diagnoses. Treatment-emergent NEPC, by contrast, develops in an estimated 15–20% of patients with castration-resistant prostate cancer (CRPC), a proportion that appears to have increased with the wider use of potent androgen receptor pathway inhibitors (ARPIs) [1,2]. In a large real-world cohort including more than 600,000 patients with prostate cancer, NEPC was identified in 0.4% of cases, with a median interval of approximately 21 months between initial prostate cancer diagnosis and NEPC detection [3]. The true burden is probably higher, as fewer than 30% of pathologically confirmed NEPC cases were labeled as NEPC in the original pathology reports [4].

The transformation from prostate adenocarcinoma to NEPC is driven by lineage plasticity, a process through which tumor cells lose dependence on androgen receptor signaling and acquire a neuroendocrine phenotype. In clinical practice, this shift often presents with low or discordant PSA levels despite radiographic or clinical progression, visceral metastases, and rapid resistance to ARPI therapy [5]. A comparable transformation is seen in EGFR-mutant lung adenocarcinoma when it converts to small-cell lung cancer (SCLC). The biological overlap between NEPC and SCLC is substantial, as both entities share neuroendocrine differentiation programs and may respond to platinum-based chemotherapy [6,7]. Current treatment strategies for NEPC are therefore still largely adapted from SCLC protocols. Outcomes remain poor, with a reported median overall survival of 9.6 months in the metastatic castration-resistant setting [4].

Because of this overlap, biomarkers already studied in SCLC may also be worth evaluating in NEPC. The Lung Immune Prognostic Index (LIPI) is a composite score based on two routinely available laboratory parameters: the derived neutrophil-to-lymphocyte ratio (dNLR) and serum lactate dehydrogenase (LDH). LIPI was first developed in patients with advanced non-small-cell lung cancer receiving immune checkpoint inhibitors, where it separated patients into three prognostic groups with median overall survival values of 16.5, 10.0, and 4.8 months [8]. Subsequent analyses of nearly 4000 patients enrolled in randomized trials showed that this prognostic association extended beyond immunotherapy and was largely independent of treatment class [9]. LIPI has since shown prognostic value in several other malignancies, including renal cell carcinoma and melanoma [10].

LIPI has also been explored within the neuroendocrine tumor spectrum. Galvano et al. were among the first to evaluate LIPI in lung neuroendocrine carcinomas, observing a survival gradient across LIPI groups, although the association did not reach statistical significance, likely because of the limited sample size [11]. More recently, a larger multicenter study in extensive-stage SCLC identified LIPI as the only independent predictor of overall survival in multivariable analysis, with a hazard ratio of 5.40 for patients in the poor LIPI group [12]. There are also biological reasons why this association may exist. Inflammatory mediators such as IL-6 and IL-8 can influence both dNLR and LDH levels and have been implicated in neuroendocrine transdifferentiation through paracrine signaling, pointing to a potential connection between systemic inflammation and the emergence of the NEPC phenotype [13].

Systemic inflammatory indices have been studied more broadly in prostate cancer as well. In a comparative analysis of seven inflammatory markers in metastatic prostate cancer, LIPI showed the strongest discriminatory ability in the hormone-sensitive setting. Its advantage was less evident in CRPC, possibly because treatments such as abiraterone can independently affect LDH levels [14]. Other inflammatory markers, including the systemic immune-inflammation index (SII) and dNLR, have also been associated with survival in large mCRPC cohorts [15,16]. However, inflammatory biomarkers have not been studied in detail in NEPC specifically. Gagnon et al. reported that elevated NLR at the time of NEPC diagnosis independently predicted mortality in 135 patients, with a hazard ratio of 1.51 [4]. LIPI, however, has not yet been evaluated in this population.

Patients with NEPC have limited prognostic tools, and a score derived from routine blood tests could help guide treatment planning without additional cost or invasive procedures. Against this background, this multicenter retrospective study aimed to assess the prognostic role of LIPI and other systemic inflammatory parameters at multiple time points in patients with NEPC, including both de novo and treatment-emergent cases.

## 2. Materials and Methods

### 2.1. Study Design and Patient Population

The study was approved by the Ethics Committee for Non-Interventional Clinical Research, Faculty of Medicine, Aydin Adnan Menderes University for on 30 April 2026 with protocol number 2026/179 and decision number 35. An additional ethics committee approval was obtained on 18 June 2026 with decision number 26. This was a multicenter retrospective study conducted across four tertiary oncology centers in Turkey: Aydın Adnan Menderes University Hospital, Pamukkale University Hospital, Antalya City Hospital, and İzmir City Hospital. Medical records were reviewed to identify patients with a confirmed diagnosis of neuroendocrine prostate cancer (NEPC).

Eligible patients were male, aged 18 years or older, with a prior diagnosis of prostate cancer followed at one of the participating centers. NEPC diagnosis required fulfillment of at least one of three criteria: (A) pathological or immunohistochemical evidence of neuroendocrine differentiation, including small-cell or large-cell neuroendocrine morphology and/or positivity for chromogranin A, synaptophysin, CD56, or INSM1; (B) radiological features consistent with neuroendocrine transformation, including predominant visceral metastases, lytic bone lesions, a mass of 5 cm or larger, or loss of PSMA expression on PSMA-PET in the setting of high-volume disease; or (C) at least two of the following four clinical features: PSA-clinical dissociation in the setting of castration-level testosterone, elevated serum chromogranin A or neuron-specific enolase, presence of a paraneoplastic syndrome, or androgen-independent progression within six months of initiating ADT or ARPI therapy. Available complete blood count and lactate dehydrogenase data at a minimum of one time point were required for inclusion.

Two distinct patient groups were defined. Patients in whom NEPC arose following a prior adenocarcinoma phase after progression through castration-resistant prostate cancer (CRPC) were classified as secondary NEPC. Those in whom NEPC was present at first presentation, without a preceding adenocarcinoma phase, were classified as de novo NEPC and analyzed as a separate subgroup.

CRPC was defined per 2024 EAU criteria as progression despite castration-level testosterone below 50 ng/dL, evidenced by serial PSA rises meeting predefined thresholds or radiological progression by RECIST 1.1.

### 2.2. Laboratory Data Collection and Time Points

Three clinical time points were defined for laboratory data collection, each with a permissible window of three months. The primary time point was NEPC diagnosis, at which laboratory data were available for all 34 patients. For secondary NEPC patients (*n* = 21), two additional time points were examined: initial prostate cancer diagnosis and CRPC diagnosis. In de novo NEPC patients (*n* = 13), the initial cancer diagnosis coincided with the NEPC diagnosis by definition.

At each available time point, the following parameters were recorded: absolute leukocyte, neutrophil, lymphocyte, monocyte, and platelet counts; serum lactate dehydrogenase (LDH); albumin; C-reactive protein; and prostate-specific antigen.

### 2.3. Inflammatory Index Calculations

The derived neutrophil-to-lymphocyte ratio (dNLR) was calculated as the absolute neutrophil count divided by the difference between total leukocyte count and absolute neutrophil count. The neutrophil-to-lymphocyte ratio (NLR), platelet-to-lymphocyte ratio (PLR), and systemic immune-inflammation index (SII = platelet count × neutrophil count/lymphocyte count) were also computed at each time point.

LIPI categories were defined using the fixed thresholds proposed by Mezquita et al. [8]. Patients with both dNLR > 3 and LDH above the institutional upper limit of normal were classified as poor risk, those with one abnormal marker as intermediate risk, and those with no abnormal markers as good risk. These thresholds were retained for consistency with the published literature. In the original scoring system, patients are classified into three prognostic categories: good (score 0), intermediate (score 1), and poor (score 2). However, given that only four patients (11.8%) in this cohort met criteria for the poor category, the intermediate and poor groups were combined into a single Intermediate + Poor group for all survival analyses. Three-group analysis with such a small poor subgroup would yield unreliable survival estimates with excessively wide confidence intervals and insufficient statistical power for meaningful interpretation.

### 2.4. Outcomes

The primary outcome was overall survival (OS), defined as the time from NEPC diagnosis to death from any cause, with patients alive at last follow-up censored at that date.

For secondary NEPC patients, two additional outcomes were examined: time from initial prostate cancer diagnosis to NEPC diagnosis, and time from CRPC diagnosis to NEPC diagnosis. These were analyzed to determine whether inflammatory indices at earlier disease stages predicted the pace of neuroendocrine transformation.

### 2.5. Statistical Analysis

Descriptive statistics are presented as median and IQR for continuous variables, and as count with percentage for categorical data. Group comparisons were performed using the Mann–Whitney U test for two-group comparisons and the Kruskal–Wallis test for three or more groups. Categorical variables were compared using the chi-square or Fisher’s exact test, as appropriate.

Survival was estimated using the Kaplan–Meier method and groups were compared with the log-rank test. Univariable and multivariable Cox proportional hazards regression were used to identify independent prognostic factors; variables with *p* < 0.10 in univariable analysis were candidates for inclusion in the multivariable model. However, NLR, dNLR, LDH, and SII were excluded from the multivariable model despite meeting this threshold, because these indices are mathematically derived from overlapping blood count components and are highly intercorrelated with LIPI, which itself incorporates dNLR and LDH. Including them simultaneously would introduce multicollinearity that invalidates model coefficients. LIPI was retained as the pre-specified primary inflammatory index of interest, and albumin was included as the only clinically independent predictor meeting the threshold. Results are expressed as hazard ratios with 95% confidence intervals. The discriminatory ability of LIPI for overall survival was assessed by receiver operating characteristic (ROC) curve analysis with calculation of the area under the curve (AUC).

All analyses were performed using SPSS version 26.0 (IBM Corp., Armonk, NY, USA). A two-sided *p*-value below 0.05 was considered statistically significant.

## 3. Results

### 3.1. Patient Characteristics

Thirty-four patients with NEPC were included from four centers. Twenty-one (61.8%) had secondary NEPC and 13 (38.2%) had de novo NEPC. All 34 patients presented with de novo metastatic disease at initial diagnosis. The median age was 66 years (IQR 60.75–72.25). ECOG performance status was 0–1 in 28 patients (82.4%) and 2–3 in 6 (17.6%). Gleason Grade Group 5 was present in 19 patients (55.9%). At NEPC diagnosis, M1c disease was most common (55.9%), followed by M1b (32.4%) and M1a (11.7%). Bone metastases were present in 85.3% of patients, visceral metastases in 55.9%, and hepatic metastases in 41.2%. High-volume disease by CHAARTED criteria was documented in 79.4% and high-risk disease by latitude in 73.5%. Pathological confirmation (Criterion A) was present in 24 patients (70.6%), radiological features (Criterion B) in 31 (91.2%), and clinical criteria (Criterion C) in 33 (97.1%), of whom 25 (73.5%) had PSA-clinical dissociation. Most patients fulfilled more than one diagnostic criterion simultaneously; three patients met Criterion C alone without pathological or radiological confirmation. Full baseline characteristics are shown in Table 1.

At NEPC diagnosis, 18 patients (52.9%) had Good LIPI and 16 (47.1%) had Intermediate or Poor LIPI; the latter two groups were combined for all analyses given the small size of the Poor subgroup (*n* = 4) (Table 1).

### 3.2. Primary Analysis: LIPI at NEPC Diagnosis and Overall Survival

**Figure 1:**
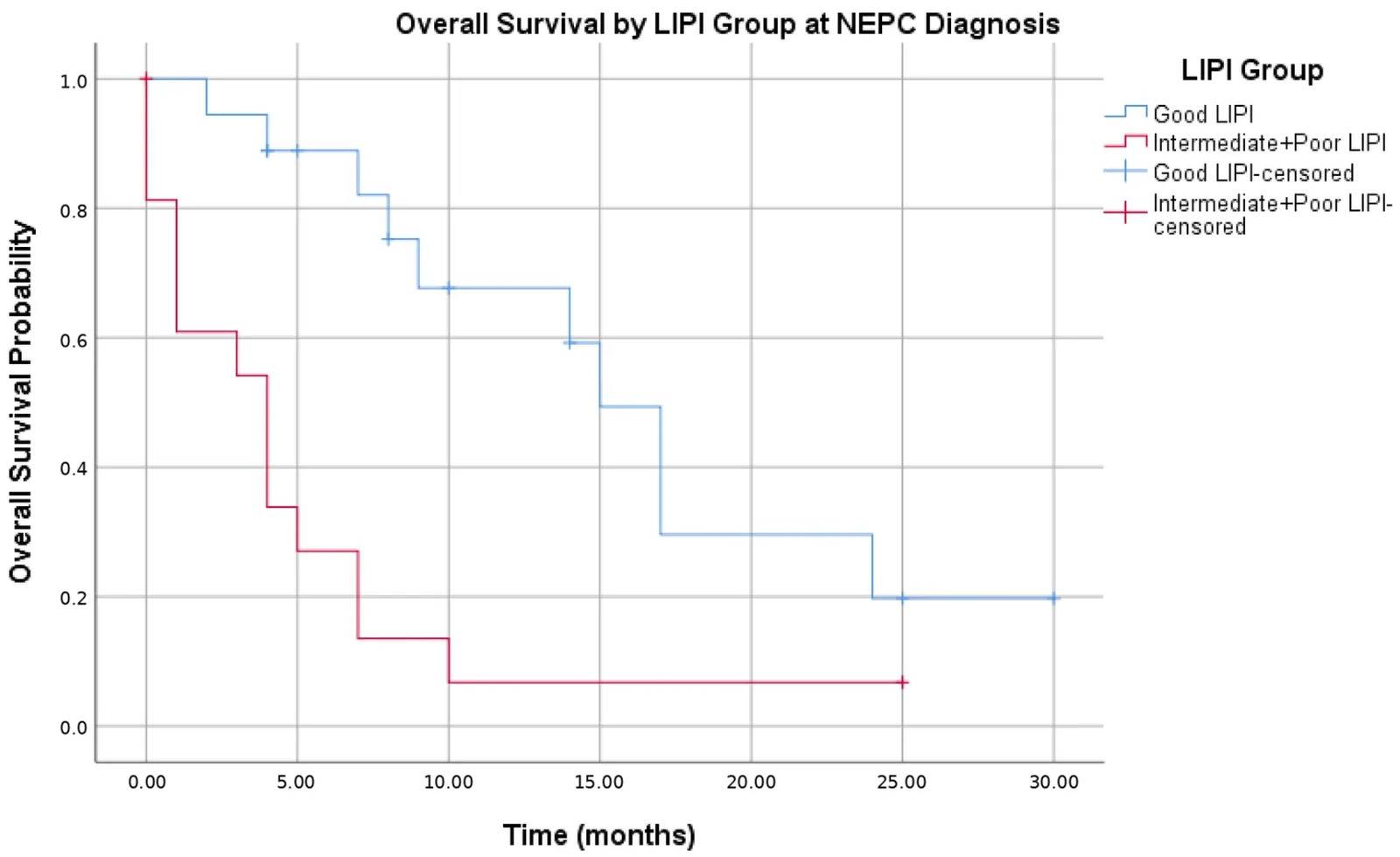
Kaplan–Meier curves for overall survival by LIPI group at NEPC diagnosis.

Patients with Intermediate + Poor LIPI at NEPC diagnosis had markedly shorter overall survival than those with Good LIPI (median 4 months vs. 15 months; log-rank *p* = 0.001) (Figure 1, Table 2).

In univariable Cox regression, Intermediate + Poor LIPI was associated with a nearly fourfold increase in mortality risk (HR 3.83, 95% CI 1.65–8.90, *p* = 0.002). Among laboratory parameters, lower albumin (HR 0.27, 95% CI 0.13–0.54, *p* < 0.001), elevated NLR (HR 1.07, *p* = 0.003), dNLR (HR 1.22, *p* = 0.003), LDH (*p* = 0.002), SII (*p* = 0.007), and PLR (*p* = 0.076) were also associated with overall survival. Age, Gleason grade group, NEPC subtype, visceral metastasis, ECOG performance status, ARPI use, and PSA at NEPC diagnosis were not significant predictors. Full univariable results are presented in Table 3.

In multivariable analysis including LIPI and albumin, albumin remained independently associated with survival (HR 0.37, 95% CI 0.17–0.83, *p* = 0.015). LIPI showed a trend that did not reach statistical significance (HR 2.35, 95% CI 0.87–6.32, *p* = 0.091) (Table 4).

**Figure 2:**
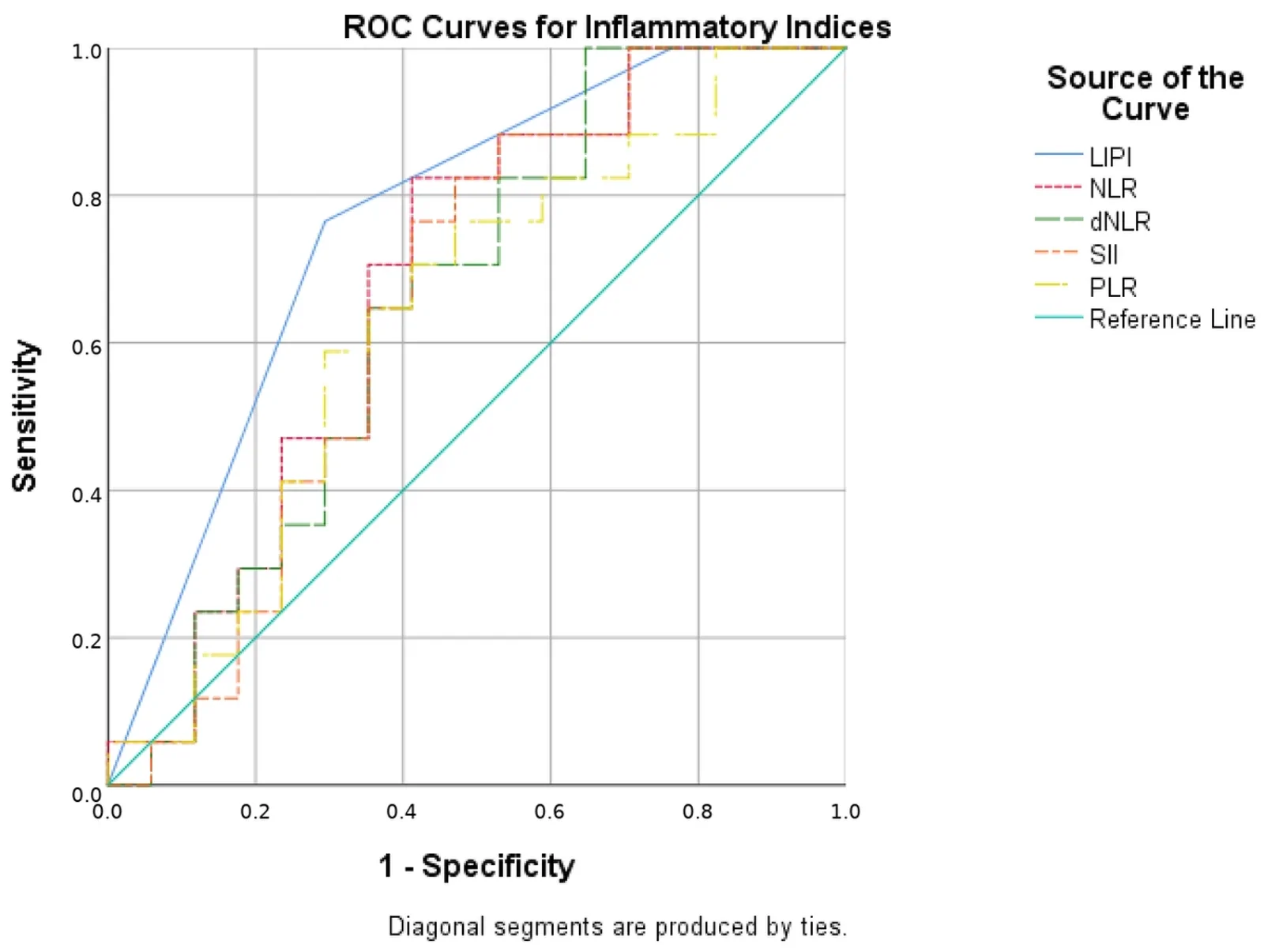
ROC curves for inflammatory indices predicting 6-month overall survival.

Among inflammatory indices, LIPI demonstrated the highest discriminatory ability for 6-month overall survival (AUC 0.763, 95% CI 0.60–0.93, *p* = 0.009), and was the only index to reach statistical significance. The AUC values for NLR (AUC 0.682, *p* = 0.071), SII (AUC 0.657, *p* = 0.117), dNLR (AUC 0.654, *p* = 0.125), and PLR (AUC 0.637, *p* = 0.174) did not reach significance. Formal pairwise AUC comparisons were not performed; the observed differences in AUC should therefore be interpreted descriptively rather than as evidence of statistically confirmed superiority (Figure 2, Table 5).

### 3.3. Secondary Analyses: LIPI at Earlier Disease Stages

Among 21 secondary NEPC patients, LIPI at initial prostate cancer diagnosis did not predict time to neuroendocrine transformation (median 25 months for Good vs. 17 months for Intermediate + Poor; HR 1.23, 95% CI 0.49–3.13, *p* = 0.660). Similarly, LIPI at CRPC diagnosis was not associated with time from CRPC to NEPC in the 16 patients with available data (median 5 months vs. 4 months; HR 0.89, 95% CI 0.30–2.63, *p* = 0.838). LIPI at initial diagnosis also did not predict time to castration resistance (median 16 months vs. 9 months; HR 1.40, 95% CI 0.55–3.56, *p* = 0.474). These results are summarized in Table 6.

ARPI use was not associated with overall survival (median 9 months for ARPI-treated vs. 7 months for untreated; *p* = 0.446). However, ARPI-treated patients showed a trend toward shorter time to castration resistance (median 12 months vs. 16 months; log-rank *p* = 0.051).

### 3.4. LIPI Trajectory and Overall Survival in Secondary NEPC

**Figure 3:**
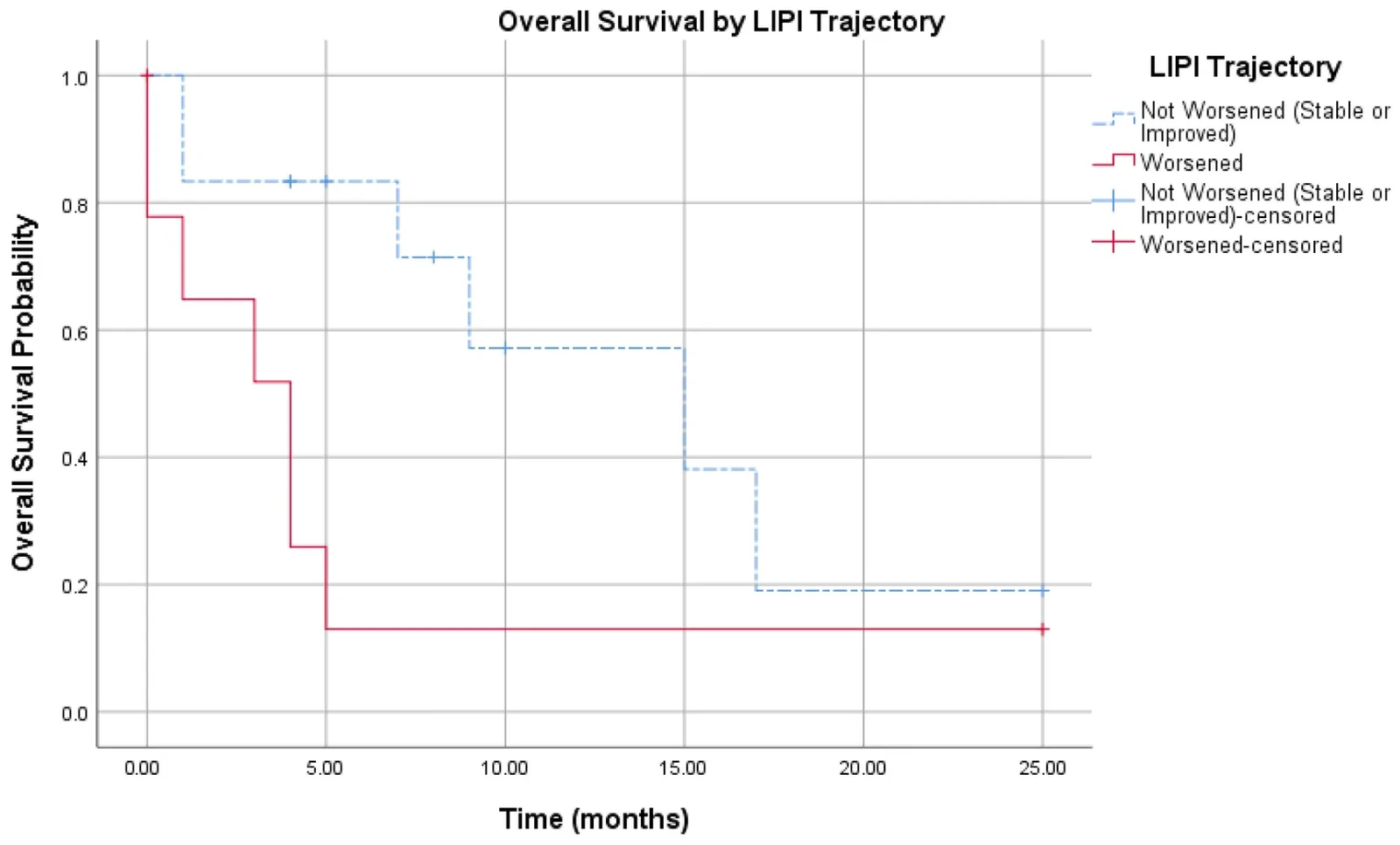
Kaplan–Meier curves for overall survival by LIPI trajectory in secondary NEPC patients.

Among 21 secondary NEPC patients, LIPI worsened from initial prostate cancer diagnosis to NEPC diagnosis in nine patients (42.9%), remained unchanged in seven (33.3%), and improved in five (23.8%). Patients whose LIPI worsened had significantly shorter overall survival than those whose LIPI did not worsen (median 4 months vs. 15 months; log-rank *p* = 0.044; HR 2.92, 95% CI 0.96–8.95, *p* = 0.060) (Figure 3, Table 6).

## 4. Discussion

In this multicenter cohort of 34 patients with neuroendocrine prostate cancer (NEPC), including both de novo and secondary subtypes, we evaluated the prognostic role of the Lung Immune Prognostic Index (LIPI). Prostate cancer is among the most common malignancies in men and remains a major cause of cancer-related mortality, with incidence increasing over recent decades in Turkey, as in many other countries [17]. NEPC is one of the most aggressive forms of the disease, yet validated prognostic biomarkers for this subgroup are still lacking. To our knowledge, this is the first multicenter study to assess LIPI specifically in patients with NEPC.

The principal finding of this analysis was that Intermediate/Poor LIPI at the time of NEPC diagnosis was associated with markedly shorter overall survival than Good LIPI. The median survival difference was 11 months, 4 months versus 15 months (log-rank *p* = 0.001; HR 3.83). Among the inflammatory indices evaluated, LIPI was the only marker that reached statistical significance for discriminating 6-month overall survival, with an AUC of 0.763 (*p* = 0.009). The only previous study focusing on inflammatory biomarkers in NEPC reported that NLR was independently associated with mortality, with a hazard ratio of 1.51 [4]. Our findings are broadly in line with that report, since NLR also showed a trend toward prognostic significance in our cohort (AUC 0.682, *p* = 0.071). The stronger performance of LIPI suggests that combining dNLR with LDH may add prognostic information beyond NLR alone. Median overall survival in our cohort was 7 months, somewhat shorter than the 9.6 months reported for metastatic castration-resistant NEPC in the previous series [4]. This difference may relate to the inclusion of de novo cases and the high proportion of patients with M1c disease in our population.

Our results also fit what has been reported for LIPI in pulmonary neuroendocrine tumors. In SCLC and LCNEC cohorts, LIPI has shown a clear survival gradient that reached significance in larger studies [11,12]. It is notable that a significant association was observed in our NEPC cohort despite the limited sample size. One possible explanation is that NEPC usually follows a very aggressive course, with short survival times that make prognostic differences more visible within a relatively brief follow-up period. Applying LIPI to prostatic neuroendocrine carcinoma is therefore not simply an extrapolation from lung cancer. It is grounded in the shared features of lineage plasticity and the continued reliance on SCLC-derived treatment protocols in NEPC management [6,7].

The relationship between LIPI and survival in NEPC can also be explained by the biology reflected in its two components. Elevated dNLR reflects systemic neutrophilia together with relative lymphopenia, a pattern often associated with impaired anti-tumor immunity and a tumor microenvironment that favors progression. LDH is commonly viewed as a marker of tumor burden and anaerobic metabolic activity. Both markers are particularly relevant in NEPC, which is characterized by rapid proliferation and often a high Ki-67 index. Inflammatory cytokines such as IL-6 and IL-8 have also been implicated in neuroendocrine transdifferentiation through paracrine signaling [13]. The inflammatory state captured by dNLR could therefore actively facilitate the neuroendocrine phenotype, rather than simply reflecting the extent of disease. By combining dNLR and LDH, LIPI reflects both systemic inflammation and tumor-related metabolic activity. This combined structure may explain its stronger performance than the individual inflammatory markers and other indices in predicting 6-month survival.

These findings can also be read alongside previous data in metastatic prostate cancer. Wang et al. reported that LIPI outperformed six other inflammatory markers in metastatic hormone-sensitive prostate cancer, although its advantage became less apparent at the CRPC stage, possibly because treatments such as abiraterone can independently influence LDH levels [14]. In our cohort, LIPI retained prognostic value even though NEPC usually develops downstream of the CRPC state. Once the neuroendocrine phenotype is established, LDH appears to regain biological relevance, likely because NEPC imposes a more aggressive and metabolically active disease pattern.

Albumin was an independent predictor of survival in multivariable analysis (HR 0.37, *p* = 0.015), whereas LIPI showed a trend that did not reach conventional statistical significance (HR 2.35, *p* = 0.091). This attenuation is most likely attributable to two interrelated factors rather than an absence of prognostic effect: the limited statistical power of a 34-patient cohort with 24 events, which is insufficient to reliably detect independent effects from correlated predictors; and the biological and statistical overlap between LIPI and albumin, as hypoalbuminemia in advanced cancer reflects the same chronic inflammatory and catabolic milieu captured by dNLR. This shift in the multivariable model is understandable. Low albumin in advanced cancer often reflects chronic inflammation, catabolic stress, and nutritional decline, all of which overlap with the inflammatory pathways represented by dNLR. In a population with rapidly progressive disease and substantial systemic inflammation, albumin may capture part of the same underlying risk reflected by LIPI. Still, this does not lessen the practical value of LIPI. As a simple, single-timepoint composite index, LIPI performed better than albumin in ROC analysis and can be calculated at NEPC diagnosis using only routine laboratory tests.

In the secondary analyses, LIPI measured at earlier disease stages was not significantly associated with time to neuroendocrine transformation or time to castration resistance. These findings should be considered in the context of the small sample size and the biology of NEPC transformation. Statistically, only 16 patients had complete laboratory data across all three time points, leaving these analyses clearly underpowered. Biologically, NEPC transformation is unlikely to be explained by a single inflammatory measurement; it is a complex process shaped by genetic, epigenetic, treatment-related, and microenvironmental changes that accumulate over time. LIPI may therefore become most informative once NEPC is already established, rather than during the earlier phases of transformation.

Among patients with secondary NEPC, those whose LIPI worsened between initial prostate cancer diagnosis and NEPC diagnosis had significantly shorter survival than those whose LIPI did not worsen. Median survival was 4 months versus 15 months (log-rank *p* = 0.044; HR 2.92, *p* = 0.060). Although the Cox model did not reach statistical significance, likely because of the limited sample size, an 11-month difference in survival is difficult to ignore in a disease with such poor outcomes. A worsening inflammatory profile over the disease course may help identify patients at especially high risk. Such patients could be considered for earlier NEPC-directed treatment or clinical trial enrollment, although this approach needs confirmation in prospective cohorts.

The exploratory comparison between de novo and secondary NEPC showed that elevated dNLR at NEPC diagnosis was more frequent in secondary cases than in de novo cases (28.6% vs. 0%, *p* = 0.034). The overall LIPI distribution, however, did not differ significantly between the two groups (*p* = 0.429). This finding should be interpreted with considerable caution. The comparison is based on six patients with elevated dNLR in the secondary group and zero in the de novo group, making the statistical result entirely dependent on small cell counts with very limited power. We present this as a preliminary observation only, and refrain from drawing biological conclusions until larger studies can address this question more reliably. ARPI use was, by definition, limited to patients with secondary NEPC, and ARPI-treated patients showed a trend toward shorter time to castration resistance (median 12 vs. 16 months; *p* = 0.051). This finding is severely limited and should not be interpreted as evidence of a causal relationship. With only seven ARPI-treated patients, the analysis is critically underpowered. More importantly, confounding by indication is a fundamental concern: patients selected for ARPI therapy likely had inherently more aggressive disease, meaning the observed difference in time to castration resistance may reflect patient selection rather than any effect of ARPI exposure. Whether ARPI-related selective pressure contributes to neuroendocrine transdifferentiation through androgen receptor-independent pathways will require prospective studies with genomic characterization.

This study has several limitations. The cohort included only 34 patients, although this is difficult to avoid given the rarity of NEPC. The study was adequately powered for the primary analysis, as the observed hazard ratio of 3.83 was detectable with 24 events in 34 patients, but the secondary and exploratory subgroup analyses conducted in 16 to 21 patients were clearly underpowered and should be interpreted as hypothesis-generating. Complete laboratory data at all three time points were available for only 16 of the 21 patients with secondary NEPC, which limited the longitudinal analyses. The retrospective design also introduces the possibility of selection bias. Centralized pathological review was not performed across the four centers, so some diagnostic heterogeneity may have been present. Tumor genomic profiling was not available, preventing us from examining how molecular subtypes may interact with systemic inflammatory patterns. Although LDH thresholds were standardized across centers, some assay variability cannot be completely excluded. Finally, CRP data were available for only 21 patients due to inconsistent collection across centers, as CRP measurement is not uniformly included in routine oncology workup at all participating institutions. Although CRP is conceptually related to systemic inflammation, it is not a component of LIPI, and conducting a sensitivity analysis restricted to 21 patients would have further reduced an already limited sample, making any conclusions unreliable. Future studies should prospectively standardize CRP collection to allow a more comprehensive evaluation of inflammatory markers in NEPC.

Despite these limitations, this study provides the first multicenter evidence that LIPI has prognostic value in NEPC. The index is practical, inexpensive, and does not require additional tissue sampling or specialized molecular testing. It can be calculated from a routine complete blood count and LDH measurement, making it useful in settings where advanced molecular profiling is not readily available. Larger prospective studies, ideally with standardized laboratory protocols, centralized pathology review, and genomic data, are needed to validate these findings and clarify how LIPI might be integrated into clinical decision-making for patients with NEPC. The prognostic landscape of prostate cancer is further shaped by genomic instability, driver gene alterations, and tumor microenvironment characteristics, as demonstrated by recent multi-omics analyses identifying aneuploidy drivers, docetaxel-resistance biomarkers, and immune microenvironment signatures in this disease [18,19,20]. Whether the prognostic value of LIPI is modified by molecular alterations associated with neuroendocrine transdifferentiation, particularly RB1 loss, TP53 mutation, and PTEN deletion, remains an open question that deserves attention in future work.

## 5. Conclusions

NEPC is an aggressive subtype of prostate cancer with limited prognostic tools at diagnosis. In this multicenter cohort, LIPI was associated with overall survival and showed better discriminatory performance for 6-month survival than individual inflammatory indices. As a low-cost and readily available score based on routine blood tests, LIPI may help support risk stratification at NEPC diagnosis. Larger prospective studies with standardized laboratory protocols and molecular data are needed to validate these findings and clarify its clinical use.

## Figures and Tables

**Table 1:** Baseline characteristics of the study population (*n* = 34).

Variable	Total Cohort (*n* = 34)
**Demographics**	
Age, years, median (IQR)	66 (60.75–72.25)
NEPC subtype	
De novo NEPC	13 (38.2)
Secondary NEPC	21 (61.8)
ECOG Performance Status	
ECOG 0–1	28 (82.4)
ECOG 2–3	6 (17.6)
Comorbidities	
Hypertension	10 (29.4)
Diabetes mellitus	7 (20.6)
Coronary artery disease	2 (5.9)
Family history of cancer	1 (2.9)
**Prostate Cancer at Diagnosis**	
Gleason Grade Group	
GG 3–4	15 (44.1)
GG 5	19 (55.9)
N stage, N1	34 (100)
M stage	
M1a	4 (11.7)
M1b	11 (32.4)
M1c	19 (55.9)
Metastatic sites at NEPC diagnosis	
Visceral metastasis	19 (55.9)
Bone metastasis	29 (85.3)
Hepatic metastasis	14 (41.2)
Pulmonary metastasis	10 (29.4)
Lymph node metastasis	34 (100.0)
CHAARTED high-volume disease	27 (79.4)
Latitude high-risk disease	25 (73.5)
**Treatment Prior to NEPC Diagnosis**	
Medical castration	34 (100)
ARPI use	7 (20.6)
ARPI type	
Enzalutamide	3 (8.8)
Abiraterone	3 (8.8)
Darolutamide	1 (2.9)
ARPI duration, months, median (IQR) †	21.5 (13.25–36.50)
**NEPC Diagnostic Criteria**	
Criterion A (Pathological/IHC)	24 (70.6)
Criterion B (Radiological)	31 (91.2)
Criterion C (Clinical/PSA dissociation) ^¶^	33 (97.1)
**NEPC Treatment**	
First-line chemotherapy regimen	
EP (etoposide + cisplatin)	24 (70.6)
EC (etoposide + carboplatin)	8 (23.5)
Other	2 (5.9)
Second-line chemotherapy	10 (32.3) ‡
**Laboratory Parameters at NEPC Diagnosis (T3)**	
Leukocytes, ×10^3^/µL, median (IQR)	7.66 (5.78–10.39)
Neutrophils, ×10^3^/µL, median (IQR)	4.79 (3.58–7.00)
Lymphocytes, ×10^3^/µL, median (IQR)	1.61 (0.88–2.30)
Monocytes, ×10^3^/µL, median (IQR)	0.49 (0.41–0.80)
Platelets, ×10^3^/µL, median (IQR)	277.5 (228.25–316.00)
dNLR, median (IQR)	2.15 (1.40–2.77)
dNLR > 3, *n* (%)	6 (17.6)
NLR, median (IQR)	3.06 (2.04–5.05)
PLR, median (IQR)	166.77 (132.86–285.23)
SII, median (IQR)	795.12 (515.63–1485.88)
LDH, U/L, median (IQR)	228.00 (177.25–412.00)
LDH > ULN, *n* (%)	14 (41.2)
Albumin, g/dL, median (IQR)	3.90 (3.04–4.20)
CRP, mg/L, median (IQR) §	9.20 (3.65–40.25)
PSA, ng/mL, median (IQR)	2.87 (0.62–65.74)
**LIPI Score at NEPC Diagnosis (T3)**	
Good (score 0)	18 (52.9)
Intermediate + Poor (score 1–2)	16 (47.1)
**Outcomes**	
Deaths, *n* (%)	24 (70.6)

Data are presented as *n* (%) for categorical variables and median (IQR) for continuous variables. All 34 patients had de novo metastatic disease at initial presentation. IQR, interquartile range; ECOG , Eastern Cooperative Oncology Group performance status; ARPI, androgen receptor pathway inhibitor; NEPC, neuroendocrine prostate cancer; EP, etoposide + cisplatin; EC, etoposide + carboplatin; dNLR, derived neutrophil-to-lymphocyte ratio; NLR, neutrophil-to-lymphocyte ratio; PLR, platelet-to-lymphocyte ratio; SII, systemic immune-inflammation index; LDH, lactate dehydrogenase; ULN, upper limit of normal; CRP, C-reactive protein; PSA, prostate-specific antigen; LIPI, Lung Immune Prognostic Index. † ARPI duration available in seven patients only. ‡ Second-line chemotherapy data available in 31 patients. § CRP available in 21 patients. LIPI Intermediate and Poor groups were merged due to small sample size in the Poor group (*n* = 4). ^¶^ Three patients met Criterion C alone without pathological or radiological confirmation.

**Table 2:** Survival estimates by LIPI group and LIPI trajectory.

Group	*n*/Events	Median OS (mo)	SE	95% CI (mo)	Min–Max (mo)	Log-Rank *p*
**Overall Survival—All Patients (** ***n*** ** = 34)**						
Overall	34/24	**7.00**	1.99	3.10–10.90	0–30	—
**By LIPI Group at NEPC Diagnosis (T3)**						
Good LIPI	18/10	**15.00**	1.44	12.17–17.83	—	**0.001**
Intermediate + Poor LIPI	16/14	**4.00**	1.36	1.34–6.66	—	—
**By LIPI Trajectory T1 → T3 (Secondary NEPC, ** ***n*** ** = 21)**						
LIPI not worsened	12/6	**10.00**	3.94	2.28–17.72	—	**0.031**
LIPI worsened	9/7	**4.00**	0.41	3.19–4.81	—	—

OS, overall survival; mo, months; SE, standard error; CI, confidence interval; LIPI, Lung Immune Prognostic Index; T1, initial prostate cancer diagnosis; T3, NEPC diagnosis. Overall survival measured from NEPC diagnosis to death from any cause. Log-rank *p* shown for the comparison between LIPI subgroups. Bold *p*-values indicate statistical significance (*p* < 0.05).

**Table 3:** Univariable Cox proportional hazards regression for overall survival.

Variable	HR	95% CI	*p*
**Inflammatory Indices**			
LIPI (Intermediate + Poor vs. Good)	3.83	1.65–8.90	**0.002**
NLR (per unit)	1.07	1.02–1.12	**0.003**
dNLR (per unit)	1.22	1.07–1.40	**0.003**
SII (per unit)	1.00	1.00–1.00	**0.007**
PLR (per unit)	1.00	1.00–1.01	**0.076**
Albumin, g/dL (per unit)	0.27	0.13–0.54	**<0.001**
**Clinical Variables**			
Age (per year)	1.03	0.98–1.09	0.247
Gleason GG5 vs. GG3–4	1.47	0.65–3.33	0.354
Secondary vs. de novo NEPC	1.01	0.45–2.27	0.978
Visceral metastasis	1.37	0.61–3.10	0.450
ECOG PS 2–3 vs. 0–1	1.97	0.77–5.01	0.155
ARPI use	0.64	0.19–2.15	0.466
PSA at NEPC diagnosis (per unit)	1.00	1.00–1.00	0.160

HR, hazard ratio; CI, confidence interval; LIPI, Lung Immune Prognostic Index; NLR, neutrophil-to-lymphocyte ratio; dNLR, derived NLR; SII, systemic immune-inflammation index; PLR, platelet-to-lymphocyte ratio; ECOG PS, Eastern Cooperative Oncology Group performance status; ARPI, androgen receptor pathway inhibitor; NEPC, neuroendocrine prostate cancer; PSA, prostate-specific antigen. Bold *p*-values indicate statistical significance (*p* < 0.05) or trend toward significance (*p* < 0.10).

**Table 4:** Multivariable Cox proportional hazards regression for overall survival.

Variable	HR	95% CI	*p*
LIPI (Intermediate + Poor vs. Good)	2.35	0.87–6.32	0.091
Albumin, g/dL (per unit)	0.37	0.17–0.83	**0.015**

HR, hazard ratio; CI, confidence interval; LIPI, Lung Immune Prognostic Index. Multivariable model included LIPI score and albumin (variables with *p* < 0.10 in univariable analysis). NLR, dNLR, LDH, and SII were excluded due to high intercorrelation with LIPI components; their simultaneous inclusion would introduce multicollinearity. Bold *p*-value indicates statistical significance (*p* < 0.05).

**Table 5:** Discriminatory ability of inflammatory indices for 6-month overall survival (ROC analysis, *n* = 34).

Inflammatory Index	AUC	95% CI	*p*
**LIPI**	**0.763**	0.60–0.93	**0.009**
NLR	0.682	0.50–0.87	0.071
SII	0.657	0.47–0.85	0.117
dNLR	0.654	0.47–0.84	0.125
PLR	0.637	0.45–0.83	0.174

AUC, area under the curve; CI, confidence interval; LIPI, Lung Immune Prognostic Index; NLR, neutrophil-to-lymphocyte ratio; SII, systemic immune-inflammation index; dNLR, derived neutrophil-to-lymphocyte ratio; PLR, platelet-to-lymphocyte ratio. Six-month overall survival was used as the binary endpoint. Bold values indicate statistical significance (*p* < 0.05).

**Table 6:** LIPI at earlier disease stages and LIPI trajectory analysis in secondary NEPC patients.

Analysis	*n*	Good LIPI Median (mo)	Int + Poor LIPI Median (mo)	Log-Rank *p*	HR	95% CI	*p*
**Secondary NEPC Subgroup—LIPI at Earlier Time Points**							
T1 LIPI → Time from PCa to NEPC diagnosis	21	25	17	0.657	1.23	0.49–3.13	0.660
T1 LIPI → Time from PCa to CRPC	21	16	9	0.462	1.40	0.55–3.56	0.474
T2 LIPI → Time from CRPC to NEPC diagnosis	16 *	5	4	0.829	0.89	0.30–2.63	0.838
**LIPI Trajectory Analysis—Secondary NEPC (** ***n*** ** = 21)**							
LIPI worsening (T1 → T3) → Overall survival	21	15 (not worsened)	4 (worsened)	**0.044**	2.92	0.96–8.95	0.060
LIPI worsening defined as any increase in LIPI score from initial prostate cancer diagnosis (T1) to NEPC diagnosis (T3). For trajectory analysis, groups: not worsened (improved or unchanged) vs. worsened.							

HR, hazard ratio; CI, confidence interval; mo, months; PCa, prostate cancer; CRPC, castration-resistant prostate cancer; NEPC, neuroendocrine prostate cancer; LIPI, Lung Immune Prognostic Index; Int + Poor, Intermediate + Poor; T1, initial prostate cancer diagnosis; T2, CRPC diagnosis; T3, NEPC diagnosis. * T2 laboratory data available in 16 of 21 secondary NEPC patients. Bold values indicate statistical significance (*p* < 0.05).

## Data Availability

The datasets generated during and/or analyzed during the current study are not publicly available due to patient privacy and institutional ethical restrictions, as the data contain identifiable clinical information from four participating centers, but are available from the corresponding author on reasonable request.
